# Insights Into the Cultivable Microbial Ecology of “Manna” Ash Products Extracted From *Fraxinus angustifolia* (*Oleaceae*) Trees in Sicily, Italy

**DOI:** 10.3389/fmicb.2019.00984

**Published:** 2019-05-21

**Authors:** Rosa Guarcello, Raimondo Gaglio, Aldo Todaro, Antonio Alfonzo, Rosario Schicchi, Fortunato Cirlincione, Giancarlo Moschetti, Nicola Francesca

**Affiliations:** Dipartimento Scienze Agrarie e Forestali, Università degli Studi di Palermo, Palermo, Italy

**Keywords:** bacteria, yeasts, filamentous fungi, microbial ecology, manna ash, osmotic environment

## Abstract

Microbial communities characterizing a specific food matrix, generally, strongly contribute to both its composition, and properties for food applications. To our knowledge, this is the first study to investigate the cultivable microbial ecology of Sicilian “Manna” ash products in order to acquire new information on the hygienic quality, shelf-life and potential application of this traditional food. To this purpose, several manna samples belonging to different commercial categories were collected and subjected to the analysis of bacteria, yeasts, and filamentous fungi. Furthermore, an investigation of the sugar content and physicochemical parameters was performed. The results of our study followed the trend generally reported for other sugary foods. Conversely, as regards microbiological analyses, in the present study, the presence of microorganisms at high levels confirmed their survival in stressing conditions characterizing this food matrix in a viable and cultivable form. Most species were osmophilic, endophytic bacteria, antagonistic of fungi pathogen of plants. Yeasts were the most abundant microbial populations and a total of six species were identified: *Candida aaseri, Candida lactis-condensi, Citeromyces matritensis, Lachancea thermotolerans, Saccharomyces cerevisiae*, and *Zygosaccharomyces bailii*. Filamentous fungi included five genera, which were considered common contaminants of honey and of other foods due to their xerophilic characteristics. Interestingly, our results suggest that the strains of *L. thermotolerans* isolated in this study might be evaluated for their potential to act as starters either singly or in multi-combination for food applications.

## Introduction

Manna is the product obtained from several species of *Fraxinus* sp. (Schicchi et al., 2007; Yücedag and Sen, 2008). During the summer season, the trunk and the main branches of the tree are notched by incision with cutters and phloem sap, a cerulean liquid, is collected. When in contact with the air, this liquid quickly thickens and forms a light crystalline whitish layer that represents manna. The dripping liquid forms a whitish stalactite of various length, in connection with the natural inclination of the trunk and the application of specific artificial tools placed on the bark (knives, metal foils, etc.). This stalactite is called “cannolo,” “manna in cannolo,” or “manna eletta” and represents the most refined part of the product. In recent times, to facilitate the formation of the cannolo, a small metal logline has been inserted under the line of the incision. The part of the sap that thickens along the trunk is instead called “manna in *rottame*,” The cultivation of ash trees in Sicily is typical of restricted areas characterized by high temperatures, low temperature range, and low air humidity, that represent the best conditions for manna production (Oieni, 1953). Current Sicilian manna production is basically obtained from *Fraxinus angustifolia* Vahl and *F. ornus* L. In recent years, the price of manna has substantially increased, becoming profitable enough to allow a recovery of the ash tree cultivation. Paradoxically, despite several new prospects available for trading in the industry of officinal biological, pharmaceutical and confectionery production, there has not yet been a significant increase in manna production. This is mainly due to the fact that the current modest production cannot meet the recent market demand which is directed to the manna in cannolo. The productions are very variable and depend on the type of product but also on the seasonal agro-climatic conditions. On average a plant in the best years produces between 3 and 4 kg of raw product.

The chemical composition of manna is very complex and variable, depending upon the species and the cultivars from which it is extracted. The most abundant active substance is the mannite or D-mannitol, a hexavalent colorless and odorless alcohol with a sugary taste, also known as “manna sugar” (Caligiani et al., 2013). Many other substances are present, such as glucose, fructose, mannotriose, mannotetraose, mineral elements, organic acids, water, and other minor components that are yet to be fully identified (Lazzarini and Lonardoni, 1984). Manna represents a pharmacologically important substance because it is employed to treat different pathologies. It is mainly used to counteract constipation problems and as a purgative with no side effects, both in childhood and adulthood (Lentini et al., 1983). In case of poisoning, the mannite produces an increase of dieresis and facilitates the removal of toxic substances in the organism through the kidneys. It is used in hypertonic solutions to remove pulmonary or cerebral edema. Manna is also recommended for the expulsion of intestinal parasites. In moderate doses, it stimulates the secretion of the biliary system. Furthermore, it is well-tolerated by diabetic patients and it can therefore be used as a food sweetener (Mazzola et al., 2016).

Manna possesses a high hygroscopic power. Therefore, it swells and undergoes processes of fermentation that are responsible for the formation of gas bubbles, and a nauseating smell of brewer's yeast (Ilardi, 1988). To our knowledge, no information is available on the microbial ecology (yeasts, bacteria, and filamentous fungi) of this product. Hence, the aim of the present work was to characterize the cultivable microorganisms associated with the several products obtained during manna processing. To this purpose, several manna samples, collected within woods located within Palermo province, were subjected to microbiological investigations. Specifically, the study included the following objectives: (i) enumeration and isolation of total bacteria, total lactic acid bacteria, osmophilic bacteria, members of *Enterobacteriaceae* family, and *Clostridium* spp., total (osmophilic and osmotolerant) yeasts and total filamentous fungi; (ii) characterization of bacterial and fungal isolates by phenotypic analysis; (iii) genotypic grouping of isolates and species identification by sequencing of specific genomic regions. The sugar content of selected samples and some physicochemical parameters were also investigated.

## Materials and Methods

### Sample Collection and Microbiological Analysis

A total of 35 manna samples (Table 1), classified as *cannolo, rottame*, and *liquid* samples, were collected from manna production throughout the Madonie area in Palermo province (37°53′N 14°01′E/37.883333°N 14.016667°E37.88333). All samples were transported to the laboratories of SAAF Department (University of Palermo) and stored in dark conditions at room temperature until analysis.

**Table 1:** Sample of manna subjected to microbiological and physic-chemical analyses.

**Sample code**	**Source**	**Site of sampling (Province of Palermo)**
1–10	Cannolo^a^	Pollina contrada Vallata
2–6	Cannolo	Castelbuono contrada Boscamento
3–8	Cannolo	Castelbuono contrada Boscamento
4–11	Cannolo	Castelbuono
5–15	Cannolo	Castelbuono “Consorzio Manna”
6–16	Cannolo	Castelbuono “Consorzio Manna”
7–10b	Cannolo	Pollina contrada Vallata
8–6b	Cannolo	Castelbuono contrada Boscamento
9–11b	Cannolo	Castelbuono
10–15b	Cannolo	Castelbuono “Consorzio Manna”
11–16b	Cannolo	Castelbuono “Consorzio Manna”
12–10c	Cannolo	Pollina contrada Vallata
13–6c	Cannolo	Castelbuono contrada Boscamento
14–11c	Cannolo	Castelbuono
15–15c	Cannolo	Castelbuono “Consorzio Manna”
16–16c	Cannolo	Castelbuono “Consorzio Manna”
17–5	Rottame^b^	Castelbuono IPSA
18–7	Rottame	Castelbuono
19–12	Rottame	Castelbuono
20–13	Rottame	Castelbuono
21–17	Rottame	Castelbuono “Consorzio Manna”
22–18	Rottame	Castelbuono “Consorzio Manna”
23–5b	Rottame	Castelbuono IPSA
24–12b	Rottame	Castelbuono
25–17b	Rottame	Castelbuono “Consorzio. Manna”
26–17b	Rottame	Castelbuono “Consorzio Manna”
27–18b	Rottame	Castelbuono “Consorzio Manna”
28–5c	Rottame	Castelbuono IPSA
29–12c	Rottame	Castelbuono
30–17c	Rottame	Castelbuono “Consorzio. Manna”
31–17	Rottame	Castelbuono “Consorzio Manna”
32–18c	Rottame	Castelbuono “Consorzio Manna”
33–9	Liquid^c^	Pollina contrada Vallata
34–9b	Liquid	Pollina contrada Vallata
35–9c	Liquid	Pollina contrada Vallata

a *Cannolo, manna sample without impurity and undamaged*.

b *Rottame, manna sample with impurity and damaged*.

c *Liquid, manna sample collected at liquid phase*.

Microbiological analyses were carried out to investigate the main microbial groups associated with manna production. Osmophilic microorganisms were counted after homogenization of samples (25 g) in a 30% (w/v) and glucose solution (sample/diluent 1:9) following the indications ISO 21527-2 to avoid shock of cells and to recover sub-lethally injured cells. The first dilution of manna samples was obtained with a stomacher (BagMixer 400, Interscience, Saint Nom, France) for 2 min at the highest speed. Cell suspensions were spread-plated and incubated as follows: total (osmophilic and osmotolerant) yeasts (TY) on tryptone glucose yeast extract agar (TGY), incubated aerobically at 25°C for 7 days (Beuchat et al., 2001); osmophilic bacteria on De Whalley Agar (DWA) supplemented with cycloeximide (170 ppm) and biphenyl (1 g/L) to inhibit the growth of yeasts and molds, incubated aerobically at 25°C for 6 days as indicated by Justé et al. (2008); osmotolerant yeasts on De Whalley Agar (DWA) supplemented with chloramphenicol (0.1 g/L) to inhibit bacteria growth, incubated aerobically at 25°C for 7 days.

All other microorganisms were recovered by homogenization of samples (25 g) in ringer solution (0.9% NaCl). Cell suspensions were plated and incubated as follows: total mesophilic count (TMC) spread on plate count agar (PCA), incubated aerobically at 30°C for 72 h; filamentous fungi (FF) spread on potato dextrose agar (PDA), incubated aerobically at 25°C for 21 d; lactic acid bacteria (LAB) poured on MRS (generic for rod LAB) and glucose (5 g/L) M17 (GM17) agar (generic for coccus LAB), incubated anaerobically with the AnaeroGen AN25 system at 30°C for 72 h; *Enterobacteriaceae* poured on violet red bile glucose agar (VRBGA), incubated aerobically by overlay agar at 37°C for 24 h; clostridia on reinforced clostridial medium (RCM) by 3 × 3 Most Probable Number (MPN) procedure (BAM, 2006).

Except VRBGA, all media used for bacterial growth were supplemented with cycloeximide (170 ppm) to inhibit the growth of yeasts and filamentous fungi, while all media used for fungal growth were supplemented with chloramphenicol (0.1 g/L) to inhibit bacterial growth. Media were purchased from Oxoid (Basingstoke, UK) and chemicals from Sigma-Aldrich (Milan, Italy). The enrichment procedure was performed for all microbial groups. All samples (10 g) were inoculated in 10 mL of different enrichment media for 48 h in relation to the same group of microorganisms: Nutrient Broth (NB) for total mesophilic microorganisms; De Whalley broth (DWB) for osmophilic bacteria (30°C); MRS broth (Oxoid, Italy) for rod LAB (30°C); M17 broth (Oxoid, Italy) for cocci LAB (30°C); YPD broth (BD Difco™, Italy) for yeasts and fungi (25°C); Buffered Peptone Water (Biolife, Italy) for *Enterobacteriaceae* (ISO 21528-1: 2017; 37°C); Cooked Meat Medium (Oxoid, Italy) for Clostryridial groups (37°C). The microbiological analyzes were conducted even after 5 days of enrichment. Microbial loads (Log CFU/g) were expressed as an average of three replicates.

### Isolation, Grouping, and Genotypic Identification of Bacteria

After growth, at least five colonies per morphology were randomly collected from the GM17 agar plates of each samples and purified to homogeneity after several sub-culturing steps onto isolation medium agar.

Presumptive *Enterobacteriaceae* and clostridia were not isolated. The isolates were purified by successive sub-culturing and the purity was checked microscopically. Presumptive LAB were phenotypically characterized by cell morphology (cocci and rods), Gram reaction (KOH method), and catalase (determined by transferring fresh colonies from a Petri dish to a glass slide and adding H_2_O_2_ 5%, v/v).

Applying the strategy described by De Angelis et al. (2001), ~30% of the isolates representing each phenotypic group for each sample were processed by RAPD analysis, with three primers (M13, AB111, and AB106) used singly by means of Thermal cycler (Swift™ MaxPro, Esco Technologies, Inc., USA). The amplified products were separated by electrophoresis, then visualized, and acquired by the KODAK Gel Logic 100 System (Kodak, Rochester, USA). The analysis of the RAPD patterns was performed with the Gelcompar II software, version 6.5 (Applied-Maths, Sint-Martens-Latem, Belgium).

One or more representative cultures for each group were identified by 16S rRNA gene sequencing. PCR reactions were performed as described by Weisburg et al. (1991) using the primers rD1 (5′-AAGGAGGTGATCCAGCC-3′) and fD1 (5′-AGAGTTTGATCCTGGCTCAG-3′). The PCR mixture (50 μ L total volume) included 62.5 ng of target DNA, 1 × Taq DNA polymerase buffer with 2 mM MgCl_2_ (ThermoFisher Scientific, Monza, Italy), 250 μM of each dNTP, 0.2 μM of each primer and 2.5 U of Taq DNA polymerase (ThermoFisher Scientific, Monza, Italy). PCR conditions were as follows: initial denaturing step at 95°C for 3 min; 30 cycles (1 min at 94°C, 45 s at 54°C, 2 min at 72°C); and an additional final chain elongation step at 72°C for 7 min. The amplicons corresponding approximately to 1,400 bp were purified using the Illustra GFX PCR DNA and Gel Band Purification Kit (GE Healthcare Bio-Sciences, Pittsburgh, PA, USA). Sequences were manually corrected and assembled using Crhomas 2.6.2. (Technelysium Pty Ltd, Australia).

The PCR products were visualized by UV transillumination on a 2% (w/v) agarose gel (Gibco BRL, Cergy Pontoise, France), stained with SYBR® Safe DNA gel stain (Molecular Probes, Eugene, OR, USA). The GeneRuler 100 bp Plus DNA Ladder (M-Medical S.r.l, Milan, Italy) was used as a molecular weight marker. The resulting DNA was sequenced by AGRIVET (University of Palermo, Italy) using the same primers employed for the PCR amplifications.The identities of the sequences were determined by BlastN search against the NCBI non-redundant sequence database located at NCBI web site^1^ http://www.ncbi.nlm.nih.gov and those of the sole type strains within the database EzTaxon, located at the EzTaxon web site^2^

### Isolation and Identification of Yeasts

As for the bacterial and yeasts characterization, five colonies were collected of different morphology with Petri dishes inoculated with the highest dilutions of each sample. Yeasts were collected for all morphologies observed on TGY and DWA-y mediums. The isolates were purified by successive sub-culturing on the same media and their purity was verified under an optical microscope (Carl Zeiss Ltd.). All isolates were subjected to overnight growth in broth media (TGY and DW broth) at the optimal temperatures. Cells were harvested, and DNA was extracted as reported by Ruzauskas et al. (2015). DNA extraction of 30% isolates was performed for the differentiation of yeasts that were obtained by RFLP of the region spanning the internal transcribed spacers (ITS1 and ITS2) and the 5.8S rRNA gene. The DNA fragments were amplified with the primer pair ITS1 (5′-TCCGTAGGTGAACCTTGCGG-3′) and ITS4 (5′-TCCTCCGCTTATTGATATGC-3′) (Esteve-Zarzoso et al., 1999) by means of T1 Thermocycler (Biometra, Göttingen, Germany) and subsequently digested with the endonucleases CfoI, HaeIII and HinfI (MBI Fermentas) at 37°C for 8 h. ITS amplicons and their restriction fragments were analyzed twice on agarose gel using at first 1.5% (w/v) agarose and then 3% (w/v) agarose in 1 × TBE buffer and visualized as reported above. Standard DNA ladders were GeneRuler 50 pb plus DNA Ladder (MMedical s.r.l., Milan, Italy). The 3% of isolate per group was further processed by sequencing the D1/D2 region of the 26S rRNA gene to confirm the preliminary identification obtained by RFLP analysis. D1/D2 region was amplified with primers NL1 (5′-GCATATCAATAAGCGGAGGAAAAG-3′) and NL4 (5′-GGTCCGTGTTTCAAGACGG-3′) (Kurtzman and Robnett, 1998). The PCR mixture (30 μ L total volume) included 100 ng of target DNA, 1 × Taq DNA polymerase buffer with 2 mM MgCl_2_ (ThermoFisher Scientific, Monza, Italy), 250 μM of each dNTP, 0.2 μM of each primer and 2.5 U of Taq DNA polymerase (ThermoFisher Scientific, Monza, Italy). PCR conditions were as follows: initial denaturing step at 95°C for 5 min; 30 cycles (1 min at 95°C, 45 s at 52°C, 1 min at 72°C); and an additional final chain elongation step at 72°C for 7 min. The amplicons corresponding ~550 bp were purified using the Illustra GFX PCR DNA and Gel Band Purification Kit (GE Healthcare Bio-Sciences, Pittsburgh, PA, USA). Sequences were manually corrected using Crhomas 2.6.2. (Technelysium Pty Ltd, Australia). The reaction of DNA sequencing and the identities of sequences were determined as reported above.

### Isolation and Identification of Filamentous Fungi

Filamentous fungi colonies were analyzed onto PDA. All colonies were identified at the genus level through observed macroscopic and microscopic characters by light microscopic analysis and dicotomous key, including color, texture, diffusible pigments, exudates, growth zones, aerial, and submerged hyphae, growth rate, and topography (Barnett and Hunter, 1998).

### Determination of Sugars

About 5 mg of samples were dissolved in 2 ml of a 1,000 ppm solution of phenyl-β-D-glucopyranoside in DMF and then sylanized with 0.5 ml TMCS and 1.0 ml HMDS. The final solution was heated at 50°C for 40 min before injection. Standard solutions of maltose, galactose, mannose, mannitol, myo-inositol, xylitol, glucose, fructose, saccharose, and gluconic acid were prepared with the same protocol. Samples were extracted with 1 ml hexane and injected (split mode) into GC–MS instrument (HP GC6890, Hewlett Packard, Palo Alto, CA) and a MS detector (HP MS5973) equipped with a Supelco SLB-5MS capillary column; (length 30 cm; internal diameter 0.25 mm; film thickness 0.25 μm) in the following chromatographic conditions: The injector temperature was 280°C. The oven starting temperature was 60°C, and after 3 min it was increased at a rate of 20°C/min until 280°C, and then held at constant temperature for 6 min. The transfer line was held at 280°C. The ion source, an electron-impact ionization (EI) type, setup at 70 eV, was held at 230°C, quadruple at 150°C, acquisition mode: scan (m/z 45–500) and calibration was done by auto-tuning. A ChemStation data system (G1701CA, Hewlett Packard, Palo Alto, CA) was used for data processing. The response factors relative to the internal standard method were calculated using the relation Ki = grI/grIS × areaIS/areai, where Ki is the response factor for the i-th species, grI is the weight of the i-th species, grIS is the weight of the internal standard, areai is the peak area of the i-th species, and areaIS is the peak area of the internal standard. All the experimental data were obtained from three replicated independent samples.

### Determination of Physicochemical Parameters

Manna samples were tested for moisture and water activity (a_w_).

Moisture content was determined by automatic moisture analyzer (Gibertini Elettronica, Novate Milanese (MI), ITALIA) at 110°C, while, Aw was determined by AquaSorp Isotherm Generator (Decagon Devices Inc., Pullman, WA, USA) at 25°C. Analyses were carried out in triplicate.

### Statistical Analysis

ANOVA test was applied to identify significant differences among the microbial counts and chemical-physical parameters. The *post-hoc* Tukey's method was applied for pairwise comparison of microbial counts and chemical-physical parameters. Statistical significance was attributed to *p* < 0.05. In addition, an explorative multivariate approach analysis by principal component analysis (PCA) has been employed, in order to investigate relationships among total sugar and bacteria/yeast population from the different manna samples.

Statistical data processing and graphic construction were performed with the XLStat software version 2014.5.03 (Addinsoft, NewYork, USA) for excel.

## Results

### Microbial Counts

The viable counts of the microbial groups examined in this study are reported in Table 2, Tables S1, S2. On PCA, the highest microbial count levels were observed in the 6–16 Manna sample (5.46 Log CFU/g). In addition, 11 samples (n. 3 of cannolo and n. 8 rottame) showed values of microbial count less than the limit of detection. The liquid manna samples (33–9, 34–9g and 35–9–1b Manna) showed TMC values in the range of 4.20–4.87 Log CFU/ml. After 48 h of incubation on PCA, 1–10 Manna sample showed TMC values of 3.48 Log CFU/ml. After 5 days, TMC values of 6.10 Log CFU/ml were detected in 17–5 Manna.

**Table 2:** Microbial loads (CFU/g or mL) of manna samples before the enrichment procedure.

**Sample code**	**Source**	**Media**							
		**Bacteria**					**Yeasts**		**FF**
		**PCA**	**GM17**	**DWA-b**	**VRBGA**	**RCM^**a**^**	**TGY**	**DWA-y**	**PDA**
1–10	Cannolo	< 2^m^	1.60 ± 0.20^def^	< 2^f^	< 1^c^	0.00	< 2^h^	< 2^k^	< 2^f^
2–6	Cannolo	2.48 ± 0.21^hijkl^	< 1^g^	< 2^f^	< 1^c^	1.39	< 2^h^	< 2^k^	< 2^f^
3–8	Cannolo	4.40 ± 0.24^bc^	1.70 ± 0.10^cdef^	2.95 ± 0.30^cd^	< 1^c^	0.00	< 2^h^	2.90 ± 0.50^ghi^	< 2^f^
4–11	Cannolo	2.00 ± 0.00^l^	< 1^g^	< 2^f^	< 1^c^	0.00	< 2^h^	< 2^k^	< 2^f^
5–15	Cannolo	2.60 ± 0.40^ghijkl^	2.00 ± 0.30^cd^	< 2^f^	< 1^c^	1.15	< 2^h^	2.48 ± 0.20^hij^	< 2^f^
6–16	Cannolo	5.46 ± 0.50^a^	2.15 ± 0.20^cd^	2.00 ± 0.00^e^	1.48 ± 0.20^a^	0.00	< 2^h^	< 2^k^	< 2^f^
7–10b	Cannolo	2.15 ± 0.12^kl^	1.60 ± 0.20^def^	< 2^f^	< 1^c^	0.00	< 2^h^	< 2^k^	< 2^f^
8–6b	Cannolo	3.47 ± 0.30^defg^	< 1^g^	< 2^f^	< 1^c^	0.00	< 2^h^	< 2^k^	< 2^f^
9–11b	Cannolo	2.11 ± 0.14^l^	1.70 ± 0.10^cdef^	< 2^f^	< 1^c^	1.21	< 2^h^	2.15 ± 0.14^ij^	< 2^f^
10–15b	Cannolo	3.26 ± 0.52^efgh^	< 1^g^	< 2^f^	1.25 ± 0.20^b^	0.00	< 2^h^	< 2^k^	< 2^f^
11–16b	Cannolo	3.09 ± 0.43^fghij^	2.00 ± 0.30^cd^	< 2^f^	< 1^c^	0.00	< 2^h^	< 2^k^	< 2^f^
12–10c	Cannolo	2.35 ± 0.35^ijkl^	2.05 ± 0.30^cd^	< 2^f^	< 1^c^	1.08	< 2^h^	< 2^k^	< 2^f^
13–6–1c	Cannolo	2.49 ± 0.46^hijkl^	2.00 ± 0.10^cd^	2.14 ± 0.10^e^	< 1^c^	0.00	< 2^h^	2.22 ± 0.13^ij^	< 2^f^
14–11c	Cannolo	< 2^m^	< 1^g^	< 2^f^	< 1^c^	0.00	< 2^h^	< 2^k^	< 2^f^
15–15c	Cannolo	< 2^m^	1.78 ± 0.30^cde^	< 2^f^	< 1^c^	0.00	< 2^h^	2.18 ± 0.10^ij^	< 2^f^
16–16c	Cannolo	2.22 ± 0.20^jkl^	1.15 ± 0.14^ef^	< 2^f^	< 1^c^	0.00	< 2	< 2^k^	< 2^f^
17–5	Rottame	< 2^m^	< 1^g^	2.00 ± 0.00^e^	< 1^c^	0.00	< 2^h^	< 2^k^	2.50 ± 0.20^d^
18–7	Rottame	2.00 ± 0.3^l^	< 1^g^	< 2^f^	< 1^c^	0.00	2.60 ± 0.30^efg^	2.00 ± 0.00^j^	< 2^f^
19–12	Rottame	3.00 ± 0.30^ghijk^	2.04 ± 0.40^cd^	2.00 ± 0.00^e^	< 1^c^	2.04	4.14 ± 0.20^bc^	4.23 ± 0.20^f^	< 2^f^
20–13	Rottame	3.18 ± 0.61^efghi^	< 1^g^	2.78 ± 0.30^d^	1.20 ± 0.10^b^	1.59	2.78 ± 0.30^def^	3.26 ± 0.40^gh^	< 2^f^
21–17	Rottame	4.04 ± 0.20^bcde^	2.86 ± 0.50^b^	2.78 ± 0.30^d^	< 1^c^	1.44	5.11 ± 0.40^a^	5.30 ± 0.10^abc^	2.00 ± 0.00^e^
22–18	Rottame	3.89 ± 0.40^cdef^	2.11 ± 0.10^cd^	3.56 ± 0.30^ab^	< 1^c^	0.00	3.30 ± 0.70^de^	4.48 ± 0.30^def^	< 2^f^
23–5b	Rottame	< 2^m^	2.31 ± 0.30^bc^	3.01 ± 0.20	< 1^c^	1.20	3.20 ± 0.10^de^	3.21 ± 0.10^gh^	< 2^f^
24–7b	Rottame	< 2^m^	1.21 ± 0.10^ef^	2.03 ± 0.02^e^	< 1^c^	0.00	4.21 ± 0.50^b^	4.28 ± 0.20^ef^	2.22 ± 0.20^de^
25–17b	Rottame	4.00 ± 0.20^bcde^	3.84 ± 0.20^a^	< 2^f^	< 1^c^	1.05	< 2^h^	5.00 ± 0.60^bcdef^	3.44 ± 0.50^ab^
26–17b	Rottame	< 2^m^	< 1^g^	< 2^f^	1.54 ± 0.10^a^	2.22	2.66 ± 0.30^efg^	< 2^k^	< 2^f^
27–18b	Rottame	3.02 ± 0.20^fghijk^	2.22 ± 0.30^bcd^	3.08 ± 0.40^bcd^	< 1^c^	1.59	3.44 ± 0.20^cd^	2.33 ± 0.10^ij^	< 2^f^
28–5–1c	Rottame	2.81 ± 0.30^ghijkl^	1.74 ± 0.40^cdef^	3.44 ± 0.20^bc^	< 1^c^	1.44	< 2^h^	3.37 ± 0.30^g^	< 2^f^
29–12d	Rottame	< 2^m^	1.10 ± 0.10^f^	< 2^f^	< 1^c^	0.00	< 2^h^	4.74 ± 0.30^cdef^	< 2^f^
30–13c	Rottame	< 2^m^	1.74 ± 0.40^cdef^	3.08 ± 0.50^bcd^	< 1^c^	0.00	5.47 ± 0.40^a^	5.11 ± 0.40^abcd^	2.13 ± 0.10^de^
31–17d	Rottame	< 2^m^	< 1^g^	3.50 ± 0.20^bc^	< 1^c^	1.33	2.01 ± 0.01^g^	5.33 ± 0.60^abc^	< 2^f^
32–18c	Rottame	< 2^m^	1.10 ± 0.10^f^	4.11 ± 0.10^a^	< 1^c^	1.40	3.31 ± 0.50^de^	< 2^k^	< 2^f^
33–9	Liquid	4.20 ± 0.50^bcd^	< 1^g^	3.30 ± 0.30^bcd^	< 1^c^	0.00	2.33 ± 0.24^fg^	5.85 ± 0.40^a^	3.80 ± 0.30^a^
34–9g	Liquid	4.52 ± 0.20^bc^	< 1^g^	2.93 ± 0.40^cd^	< 1^c^	0.00	4.97 ± 0.30^a^	5.76 ± 0.10^ab^	2.97 ± 0.10^c^
35–9–1b	Liquid	4.87 ± 0.30^ab^	< 1^g^	3.21 ± 0.10^bcd^	< 1^c^	0.00	5.25 ± 0.20^a^	5.04 ± 0.30^bcde^	3.22 ± 0.40^bc^
Statistical significance^b^		^***^	^***^	^***^	^**^		^***^	^***^	^***^

a *As estimated by MPN. FF, filamentous fungi; PCA, plate count agar for total mesophilic bacteria; GM17, glucose M17 added with cycloeximide for lactic acid bacteria counts; DWA-b, De Whalley agar added with cycloeximide for osmophilic bacteria counts; VRBGA, violet red bile glucose agar for Enterobacteriaceae counts; RCM, reinforced clostridial medium; TGY, tryptone glucose yeast extract agar added with chloramphenicol for total (osmophilic and osmotolerant) yeasts; DWA-y, De Whalley agar added with chloramphenicol for osmotolerant yeast counts; PDA, potato dextrose agar for filamentous fungi counts. Results indicate mean values with standard deviation. Data within a column followed by the same letter are not significantly different according to Tukey's test*.

b *P-value*:

*** *P < 0.001*;

** *P < 0.01*.

The highest microbial count values of LAB populations were observed in the 25–17b Manna sample (3.84 Lof CFU/g). A total of 13 samples (n. 5 cannolo, n. 5 rottame and n. 3 liquid) showed values lower than the limit of detectability and then underwent an enrichment procedure. The enrichment procedure after 48 h of incubation allowed for detection of the LAB at levels between 1.22 and 5.70 Log CFU/ml. No LAB was found after 5 days of incubation throughout the enrichment procedure.

The microbial count values of osmophilic bacteria were between 2.00 and 4.11 Log CFU/g or ml. Seventeen samples showed values lower than the limit of detection and after enrichment procedure for 48 h, 10 samples (n. 9 from cannolo and n. 1 from rottame) showed in DWA-b values in the range of 2.12–5.69 Log CFU/ml. Seven manna samples were negative due to the presence of osmophilic bacteria after 5 days of enrichment.

The presence of *Enterobacteriaceae* was detected in four samples (6–16, 10–15b, 20–13, and 26–17b Manna) with microbial count values in the range of 1.20–1.54 Log CFU/g. The presence of presumptive *Clostridium* spp. on RCM medium was observed in n. 4 samples of manna from cannolo and n. 9 samples of manna from rottame. The enrichment procedures did not increase the concentration of *Enterobacteriaceae* and Clostridial group in all samples after both 2 and 5 days of incubation.

The levels of detected yeast populations were different in relation to the source of the samples and the culture medium used. Before the enrichment procedure, all the samples coming from cannolo in TGY (n. 17) showed values lower than the detection limit. While 12 samples of manna from scrap showed total yeast (TY) levels in the range of 2.01–5.47 CFU/g. The presence of TY in the liquid manna samples in TGY was between 2 and 5 logarithmic cycles. After 48 h of incubation through an enrichment procedure, all the cannolo samples, except the 7–10b and 14–11c samples, showed microbial count levels between 2.20 and 8.04 Log CFU/ml. The presence of osmotolerant yeast count has reached higher levels (2.00–5.85 Log CFU/g or ml) in samples from rottame and liquids. While the cannolo manna samples showed a microbial concentration around two logarithmic cycles. After the enrichment procedure, of the 14 samples (11 cannoli and 3 scrap) only four samples showed values lower than the detection limit. The enrichment procedure up to 5 days allowed to obtain osmotolerant yeasts only in the sample 1–10 on DWA-y (8.25 Log CFU/ml).

### Isolation and Identification of Bacteria

**Figure 1:**
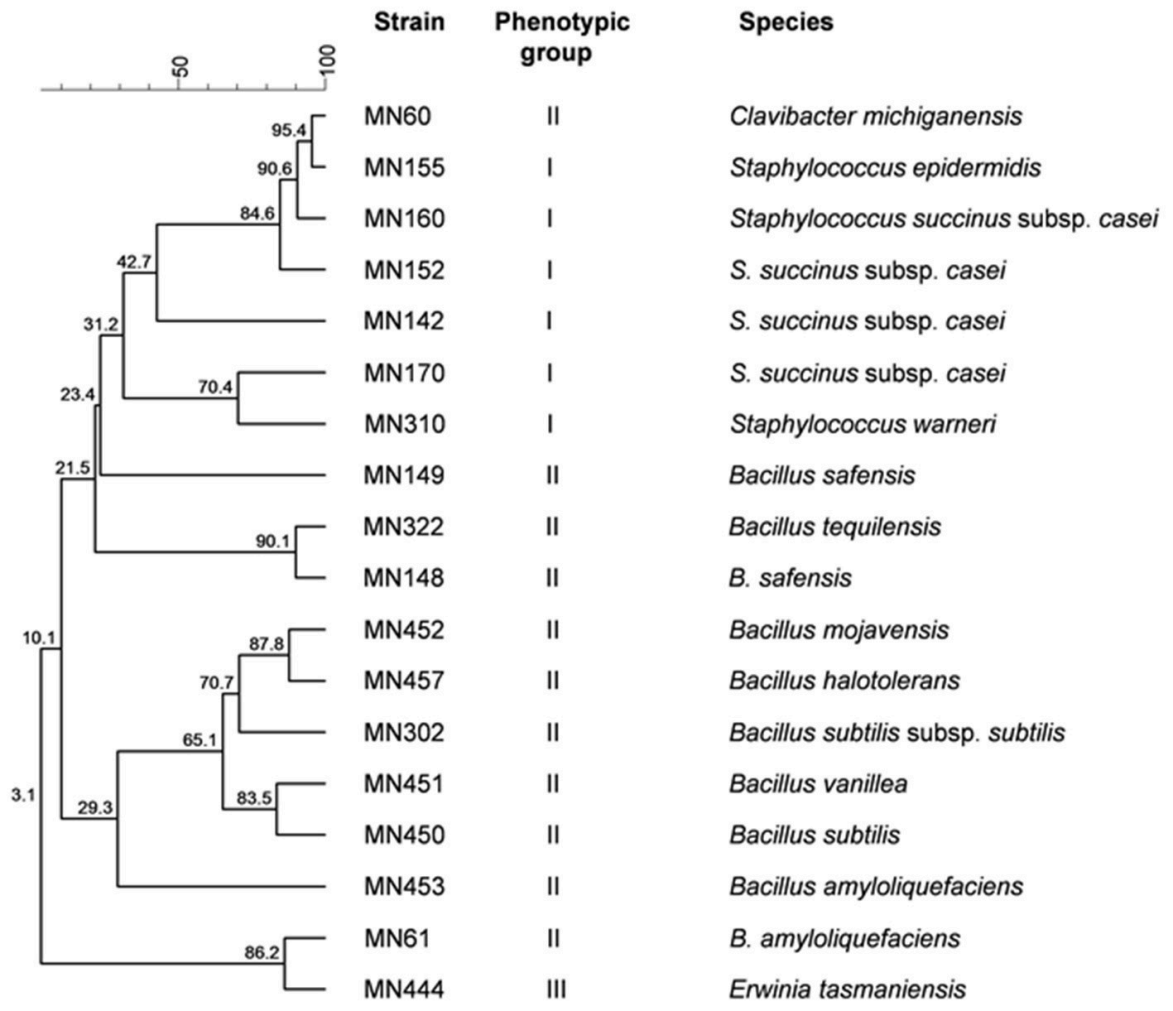
Dendrogram of bacterial strains obtained with combined RAPD-PCR patterns with primers M13, AB106, and AB111.

A total of 3,175 pure cultures were isolated and purified from count plates, specifically 1,505 isolates were obtained on DWA-b, while 1,670 isolates had come from GM17. After the Gram characterization and catalase test, no presumptive LAB were found within the isolates from GM17. In MRS, all the manna samples that were also subjected to enrichment procedure showed values of microbial counts that were lower than the limits of detection. The phenotypic group of bacteria isolates from manna samples were reported in Table 3. The results of the identification process are better detailed in Table 4. About 30% of the isolates sharing the phenotypic characteristics were analyzed by RAPD-PCR. As reported in the dendrogram (Figure 1), the cultivable bacterial community associated to the different manna samples collected. All 18 strains were identified by sequencing of the 16S rRNA gene and were deposited in GenBank (Acc. No.MK509926–MK509943). The bacteria were represented by 11 species: *Bacillus amyloliquefaciens, Bacillus halotolerans, Bacillus mojavensis, Bacillus safensis, Bacillus subtilis, Bacillus tequilensis, Bacillus vanillea, Clavibacter michiganensis, Erwinia tasmaniensis Staphylococcus epidermidis, Staphylococcus succinus* subsp. *Casei*, and *Staphylococcus warneri*.

**Table 3:** Phenotypic grouping of bacteria isolated from manna samples.

**Characteristic**	**Phenotype of cluster**		
	**I (*n* = 2,291)**	**II (*n* = 837)**	**III (*n* = 47)**
Morphology	Rods	Cocci	Rods
Gram	+	+	–
Catalase	+	+	+

**Table 4:** Molecular identification by PCR amplified products of 16S rDNA of manna bacteria strains.

**Manna source**	**Species**	**% similarity (accession no. of closest relative) by**		**Sequence lenght (bp)**	**Acc. No**.
		**BLAST**	**EzTaxon**		
Rottame	*Bacillus safensis*	99 (NR_148787.1)	99.66 (FO-36b)	1480	MK509926
Rottame	*B. safensis*	99 (NR_113945.1)	99% (FO-36b)	1477	MK509927
Cannolo	*Staphylococcus succinus* subsp. *casei*	99 (NR_037053.1)	99.45 (SB72)	1480	MK509928
Rottame	*Clavibacter michiganensis*	98% (NR_152027.1)	99.79 (LPPA 982)	1423	MK509929
Cannolo	*Bacillus amyloliquefaciens*	99 (NR_112685.1)	99.86 (DSM 7)	1449	MK509930
Cannolo	*Staphylococcus epidermidis*	99 (NR_113957.1)	99.32 (NCTC 11047)	1477	MK509931
Liquid	*S. succinus* subsp*. casei*	99 (NR_037053.1)	99.31 (SB72)	1476	MK509932
Cannolo	*Staphylococcus warneri*	99 (NR_025922.1)	99.66 (ATCC 27836)	1477	MK509933
Rottame	*Bacillus mojavensis*	100 (NR_112725.1)	99.93 (RO-H-1)	1436	MK509934
Rottame	*B. amyloliquefaciens*	99 (NR_112685.1)	99.38 (DSM 7)	1478	MK509935
Rottame	*Bacillus halotolerans*	99 (NR_115063.1)	99.72 (ATCC 25096)	1447	MK509936
Rottame	*St. succinus* subsp. *casei*	99 (NR 037053.1)	99.79 (SB72)	1449	MK509937
Rottame	*St. succinus* subsp. *casei*	99 (NR_037053.1)	99.79 (SB72)	1438	MK509938
Rottame	*Bacillus subtilis* subsp. *subtilis*	99 (NR_102783.2)	99.72 (NCIB 3610)	1435	MK509939
Cannolo	*Bacillus tequilensis*	99 (NR_104919.1)	100 (KCTC 13622)	1457	MK509940
Liquid	*Erwinia tasmaniensis*	99 (NR_074869.1)	98.81 (Et1/99)	1427	MK509941
Rottame	*Bacillus vanillea*	99 (KF986320.1)	99 [XY18(T)]	1363	MK509942
Rottame	*B. subtilis*	99 (NR_113265.1)	99.73 (KCTC 13429)	1473	MK509943

### Isolation and Identification of Yeasts and Molds

A total of 2,888 pure cultures were isolated and purified from count plates for yeasts on TGY (n. 1238) and DWA-y (n. 1650). Eight hundred and seventy isolates were clustered into 10 groups according to the RFLP profile. The genetic analysis allowed the identification of the following species: *Candida aaseri* (*n* = 54), *Candida lactis-condensi* (*n* = 72), *Citeromyces matritensis* (*n* = 67)*, Lachancea termotolerans* (*n* = 616), *Saccharomyces cerevisiae* (*n* = 33), and *Zygosaccharomyces bailii* (*n* = 28). The majority of yeasts belonged to the species *Lachancea termotolerans* (Table 5).

**Table 5:** Length in pb of the PCR amplified products of 5.8S rDNA-ITS region, ITS-RFLP, and identified species of yeast isolated from manna samples.

**Manna Source**	**Size amplicons 5.8S-ITS (bp)**	**Size of restriction fragment**^**a**^			**Species**	**% similarity (accession no. of closest relative) by BLAST^**b**^**	**Sequence lenght (bp)**	**Acc. No**.
		***Cfo*I**	***Hae*III**	***Hinf*I**				
Rottame	680	260 + 240	300 + 260 + 120	275	*Lachancea termotolerans*	100 (XR 002432231.1)	531	MK513712
Rottame	790	260 + 240	700	275	*Zygosaccharomyces bailii*	100 (NG 055054.1)	554	MK513713
Rottame	680	260 + 240	300 + 260 + 120	275	*L. termotolerans*	100 (XR 002432231.1)	543	MK513714
Cannolo	680	260 + 240	300 + 260 + 120	275	*L. termotolerans*	100 (XR 002432231.1)	540	MK513715
Cannolo	680	260 + 240	300 + 260 +120	275	*L. termotolerans*	100 (XR 002432231.1)	540	MK513716
Rottame	800	260 + 240	300 + 260 + 120	275	*L. termotolerans*	100 (XR 002432231.1)	525	MK513717
Rottame	700	320 + 280	n.c.	290 +265	*Citeromyces matritensis*	97 (KY 106923.1)	555	MK513718
Rottame	700	320 + 280	n.c.	290 +265	*C. matritensis*	99 (KY 106921.1)	555	MK513719
Rottame	680	260 + 240	300 + 260 + 120	275	*L. termotolerans*	100 (DQ 655683.1)	553	MK513720
Rottame	880	250 + 230	n.c.	260	*Saccharomyces cerevisiae*	99 (KY 471391.1)	552	MK513721
Cannolo	680	n.c.	550	285	*Candida aaseri*	100 (EU807900.1)	496	MK513722
Cannolo	600	n.c.	550	285	*C. aaseri*	100 (KY 106270.1)	490	MK513723
Cannolo	680	210 + 200	600	275	*L. termotolerans*	100 (DQ 655683.1)	542	MK513724
Cannolo	680	210 + 200	600	275	*L. termotolerans*	100 (DQ 655683.1)	548	MK513725
Rottame	680	590	600	275	*L. termotolerans*	100 (KT922994.1)	529	MK513726
Rottame	680	210 + 200	n.c.	275	*L. termotolerans*	100 (KT922390.1)	547	MK513727
Cannolo	680	210 + 200	600	275	*L. termotolerans*	100 (KT922994.1)	543	MK513728
Rottame	680	210 + 200	n.c.	275	*L. termotolerans*	100 (KT922994.1)	548	MK513729
Rottame	680	210 + 200	n.c.	275	*L. termotolerans*	100 (KT922994.1)	546	MK513730
Rottame	680	320 + 280	n.c.	275	*L. termotolerans*	100 (KT922994.1)	541	MK513731
Cannolo	680	320 + 280	n.c.	275	*L. termotolerans*	100 (KT922994.1)	533	MK513732
Liquid	680	320 + 280	n.c.	275	*L. termotolerans*	99 (KT922994.1)	543	MK513733
Rottame	680	320 + 280	n.c.	275	*L. termotolerans*	99 (KT922994.1)	545	MK513734
Rottame	680	320 + 280	n.c.	275	*L. termotolerans*	100 (KT922994.1)	542	MK513735
Rottame	680	320 + 280	n.c	275	*L. termotolerans*	100 (KT922994.1)	546	MK513736
Cannolo	680	320 + 280	n.c.	275	*L. termotolerans*	100 (KT922994.1)	553	MK513737
Rottame	700	320 + 280	n.c.	290 + 265	*C. matritensis*	100 (EF550346.1)	579	MK513738
Liquid	500	175 + 100	300	180 + 170	*Candida lactis-condensi*	100 (JN248611.1)	463	MK513739
Cannolo	500	175 + 100	300	180 + 170	*C. lactis-condensi*	100 (JN248611.1)	462	MK513740

a *Values refer to the number of base pairs per fragment*.

b *Results of Blast comparison of the D1/D2 region of the 26S rRNA gene into Genbank database*.

With regards to the filamentous fungi obtained from manna just after the collection and enrichment, based on the morphological traits, five main groups (genera) were recognized: *Alternaria, Aspergillus, Cladosporium, Mucor*, and *Penicillium*. The distribution of filamentous fungi among samples are reported in Table 6. A total of 251 filamentous fungus isolates were obtained. The most representative genus is *Cladosporium* spp. with 95 isolates.

**Table 6:** Genus of filamentous fungi.

**Code**	***Cladosporium*** **spp**.		***Mucor*** **spp**.		***Aspergillus*** **spp**.		***Alternaria*** **spp**.		***Penicillium*** **spp**.	
1–10		•		•				•		•
16–16c		•				•				•
17–5	□									
21–17	□									
24–7b	□									
25–17b	□									
30–13c	□									
31–17d		•		•		•		•		
33–9	□									
34–9	□									
35–9–1b	□									

*□ Manna just after the collection*.

*• Manna after 48 h of enrichment*.

### Sugar Content and Physicochemical Parameters

The results of the sugar content, aw and RH% of the manna samples are shown in Table 7.

**Table 7:** Physicochemical parameters of manna samples.

**Sample code**	**Source**	**Mannitol**	**Sorbitol**	**Myoiositol**	**Fructose**	**Glucose**	**Galactose**	**Mannose**	**Saccharose**	**Mannotriose**	**Stachiose**	**UR%**	**a_**w**_**
1–10	Cannolo	48.07^efgh^	0.55^efghi^	0.17^klm^	16.47^i^	2.47^pq^	0.07^lm^	0.14^ef^	0.78^cde^	23.26^e^	3.80^q^	7.47^d^	0.595^bcd^
2–6	Cannolo	51.12^b^	0.73^cd^	0.05^nop^	9.12^rs^	2.81^kl^	0.01^m^	0.02^ijk^	0.02^k^	16.48^ij^	1.81^rs^	8.33^c^	0.744^abc^
3–8	Cannolo	60.27^a^	0.89^ab^	0.03^op^	8.76^st^	3.02^hi^	0.00^m^	0.00^k^	0.00^k^	15.34^lm^	1.90^r^	11.03^a^	0.801^a^
4–11	Cannolo	47.25^ghi^	0.54^fghi^	0.22^hijk^	9.67^pqr^	2.16^st^	0.72^ghi^	0.01^jk^	0.49^gh^	25.32^bc^	10.05^g^	4.48^kl^	0.569^bcd^
5–15	Cannolo	33.48^nop^	0.51^ghij^	0.51^b^	23.22^e^	4.51^b^	1.21^b^	0.37^c^	0.76^cde^	15.26^lm^	11.53^d^	4.63^jk^	0.554^bcd^
6–16	Cannolo	48.65^defg^	0.58^defgh^	0.18^jk^	9.74^pq^	2.22^s^	0.70^ghi^	0.00^k^	0.75^de^	25.12^cd^	9.87^g^	3.19^q^	0.539^d^
7–10b	Cannolo	50.86^b^	0.74^bc^	0.04^op^	8.98^st^	2.65^mn^	0.01^m^	0.01^jk^	0.00^k^	15.46^klm^	1.75^rs^	10.64^b^	0.754^ab^
8–6b	Cannolo	46.21^i^	0.61^cdefgh^	0.24^ghij^	10.11^op^	2.42^qr^	0.76^fgh^	0.01^jk^	1.01^b^	24.63^d^	7.11^l^	2.99^r^	0.609^abcd^
9–11b	Cannolo	48.96^cdef^	0.58^defgh^	0.11^mn^	9.05^st^	3.22^g^	0.25^k^	0.12^efgh^	0.07^k^	12.04^s^	6.01^n^	0.57^v^	0.520^d^
10–15b	Cannolo	48.78^def^	0.67^cdef^	0.05^nop^	11.34^m^	2.88^jk^	0.00^m^	0.00^k^	0.00^k^	14.48^no^	1.67^s^	6.42^ef^	0.548^cd^
11–16b	Cannolo	46.82^hi^	0.73^cd^	0.25^fghi^	10.56^no^	2.59^no^	0.67^ghi^	0.00^k^	0.54^fgh^	26.49^a^	6.36^m^	6.55^e^	0.535^d^
12–10c	Cannolo	49.11^cde^	0.70^cde^	0.04^op^	12.45^kl^	2.93^ij^	0.01^m^	0.02^ijk^	0.00^k^	13.33^q^	1.70^rs^	5.39^h^	0.541^cd^
13–6c	Cannolo	48.88^def^	0.91^a^	0.03^op^	12.12^kl^	2.72^lm^	0.01^m^	0.00^k^	0.00^k^	14.29^op^	1.75^rs^	5.39^h^	0.585^bcd^
14–11c	Cannolo	50.34^bc^	0.92^a^	0.04^op^	12.54^k^	2.76^l^	0.00^m^	0.02^ijk^	0.01^k^	13.82^pq^	1.82^rs^	3.14^qr^	0.598^abcd^
15–15c	Cannolo	49.76^bcd^	0.63^cdefg^	0.19^ijk^	11.02^mn^	2.56^nop^	0.78^efg^	0.00^k^	1.21^a^	23.78^e^	9.98^g^	4.44^l^	0.553^bcd^
16–16c	Cannolo	47.57^fghi^	0.48^ghijkl^	0.18^jkl^	9.11^st^	2.06^t^	0.68^ghi^	0.00^k^	0.69^ef^	25.69^b^	8.47^j^	4.46^kl^	0.549^cd^
17–5	Rottame	37.33^jk^	0.35^klmno^	0.34^d^	22.48^f^	3.65^f^	1.19^b^	0.35^c^	0.89^bcd^	16.71^hi^	11.07^e^	3.28^q^	0.578^bcd^
18–7	Rottame	36.17^kl^	0.37^jklmn^	0.32^de^	21.22^g^	3.78^e^	1.09^bcd^	0.33^c^	0.94^bc^	15.98jk	11.13^e^	5.83^g^	0.561^bcd^
19–12	Rottame	30.21^rs^	0.41^ijklm^	0.00^p^	4.41^v^	1.54^v^	0.00^m^	0.00^k^	0.00^k^	8.12^v^	0.87^t^	2.28^s^	0.545^cd^
20–13	Rottame	34.07^mno^	0.19^pq^	0.31^def^	20.93^g^	5.13^a^	0.93^def^	0.06^hijk^	0.42^hi^	9.06^u^	11.68^d^	1.43^u^	0.573^bcd^
21–17	Rottame	35.17^lm^	0.60^cdefgh^	0.04^op^	15.76^j^	1.65^u^	0.41^jk^	0.70^a^	0.93^bcd^	10.63^t^	8.84^i^	6.26^f^	0.545^cd^
22–18	Rottame	35.31^lm^	0.19^pq^	0.44^c^	9.15^rs^	1.72^u^	1.17^bc^	0.39^c^	0.44^hi^	14.21^op^	14.42^a^	2.00^t^	0.574^bcd^
23–5b	Rottame	32.98^opq^	0.33^lmnop^	0.65^a^	11.94^l^	3.65^f^	0.71^ghi^	0.59^b^	0.63^efg^	6.50^x^	9.13^h^	1.31^u^	0.546^cd^
24–12b	Rottame	34.47^mn^	0.36^jklmno^	0.06^nop^	25.85^c^	4.40^c^	1.04^bcd^	0.35^c^	0.50^gh^	15.20^lm^	4.27^p^	3.88^n^	0.562^bcd^
25–17b	Rottame	36.08^kl^	0.41^ijklm^	0.31^def^	27.12^b^	4.16^d^	1.16^bc^	0.65^ab^	0.78^cde^	12.60^r^	0.11^u^	3.21^q^	0.563^bcd^
26–17b	Rottame	28.06^t^	0.30^mnopq^	0.08^no^	9.23^qrs^	3.00^i^	1.43^a^	0.64^ab^	0.06^k^	15.70^k^	3.87^q^	4.07^m^	0.537^d^
27–18b	Rottame	34.92^lmn^	0.19^pq^	0.01^p^	7.12^u^	3.25^g^	0.56^hij^	0.08^fghi^	0.28^ij^	13.36^q^	13.87^b^	4.34^l^	0.581^bcd^
28–5c	Rottame	35.92^kl^	0.50^ghijk^	0.27^efgh^	3.66^w^	2.14^st^	1.19^b^	0.07^ghij^	1.02^b^	15.50^kl^	9.31^h^	3.82^no^	0.578^bcd^
29–12c	Rottame	33.49^nop^	0.35^klmno^	0.30^defg^	8.56^t^	2.15^st^	0.68^b^	0.13^efg^	0.45^ghi^	19.28^g^	13.03^c^	3.69^op^	0.556^bcd^
30–17c	Rottame	29.54^s^	0.24^nopq^	0.31^def^	33.64^a^	3.11^h^	0.66^ghi^	0.35^c^	0.12^jk^	16.68^hi^	4.78^o^	4.76^ij^	0.552^bcd^
31–17	Rottame	31.63^qr^	0.21^opq^	0.34^d^	24.03^d^	2.52^opq^	1.06^bcd^	0.25^d^	0.45^ghi^	14.93^mn^	10.27^f^	7.42^d^	0.550^bcd^
32–18c	Rottame	32.24^pq^	0.49^ghijk^	0.23^hijk^	19.35^h^	3.81^e^	1.19^b^	0.15^e^	0.48^gh^	21.46^f^	3.81^q^	4.91^i^	0.547^cd^
33–9	Liquid	38.72^j^	0.47^hijkl^	0.51^b^	16.45^i^	0.88^w^	0.65^ghi^	0.06^hijk^	0.93b^cd^	17.09^h^	7.36^k^	7.49^d^	0.574^bcd^
34–9b	Liquid	32.20^pq^	0.16^q^	0.02^op^	33.49^a^	2.35^r^	0.54^ij^	0.38^c^	0.10^jk^	10.50^t^	8.38^j^	3.64^p^	0.556^bcd^
35–9c	Liquid	38.00^j^	0.17^q^	0.16^lm^	23.19^e^	3.82^e^	0.97^cde^	0.39^c^	0.36^hi^	7.31^w^	8.84^i^	3.90^mn^	0.525^d^
S.E.		1.40	0.04	0.03	1.32	0.15	0.08	0.04	0.06	0.92	0.71	0.40	0.01
Statistical significance		^***^	^*^	^*^	^***^	^**^	^*^	^*^	^*^	^***^	^***^	^***^	^*^

*UR, relative humidity; a_w_, water activity; S.E., standard error*.

*The sugar content is expressed as g/100 g*.

*Data within a columns followed by the same letter are not significantly different according to Tukey's test*.

* *P < 0.05*;

** *P < 0.01*;

*** *P < 0.001*.

Globally, the quali-quantitative sugar profile of the samples included mannitol, mannotriose, fructose, stachyose, and glucose as the most abundant saccharides.

In the manna samples classified as cannolo, the sugars present in greater quantities were mannitol and mannotriose. Samples 3–8 showed the highest values of mannitol (60.27/100 g), while the highest level of mannotriose was obtained in the 11–16b samples (26.49/100 g).

Similar situation was observed in the manna samples coming from the rottame. In particular, an analysis of average values showed that the sugar composition is mainly represented by mannitol (28.06–37.33/100 g) and fructose (3.66–33.64/100 g). A similar situation was observed for the liquid manna samples.

Globally, the UR% and a_w_ values ranged between 0.57–11.03 and 0.520–0.801, respectively. The highest values of a_w_ (0.801) and UR% (11.03) e were observed in samples 3–8.

**Figure 2:**
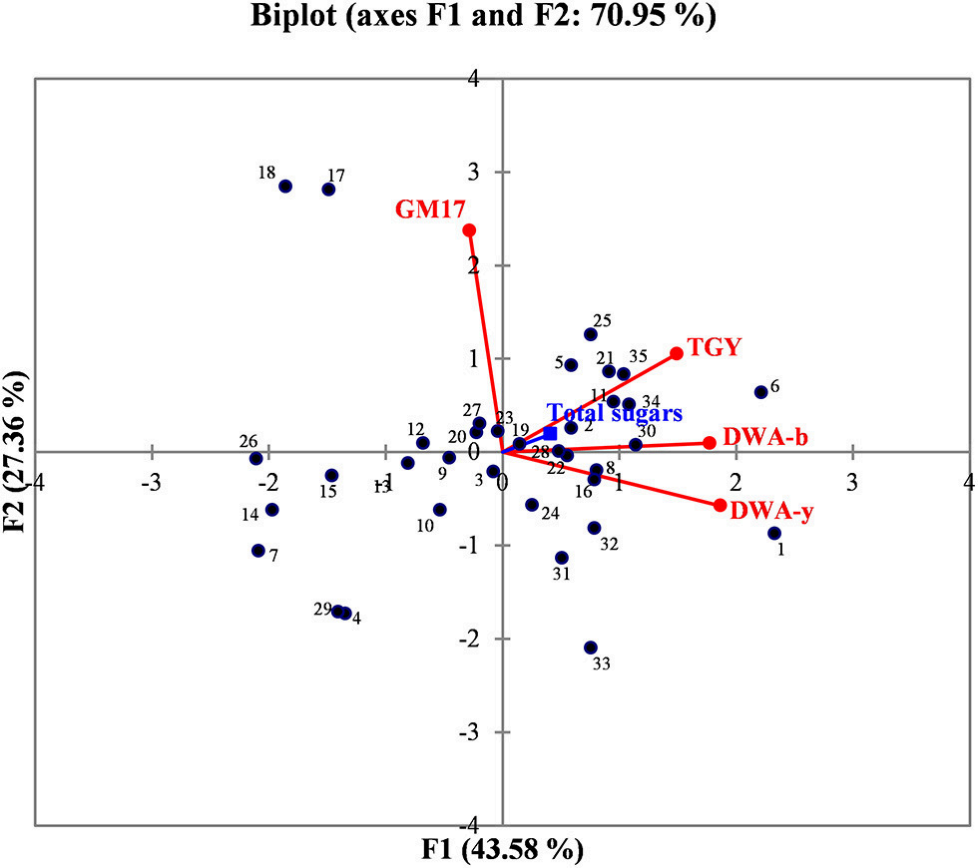
Biplot graph showing the distribution of manna samples in relation to the content of total sugar, bacteria, and yeast contaminations. GM17, glucose M17 added with cycloeximide for lactic acid bacteria counts; DWA-b, De Whalley agar added with cycloeximide for osmophilic bacteria counts; TGY, tryptone glucose yeast extract agar added with chloramphenicol for total (osmophilic and osmotolerant) yeasts; DWA-y, De Whalley agar added with chloramphenicol for osmotolerant yeast counts. Numbers in the biplot: 1, 1–10; 2, 2–6; 3, 3–8; 4, 4–11; 5, 5–15; 6, 6–16; 7, 7–10b; 8, 8–6b; 9, 9–11b; 10, 10–15b; 11, 11–16b; 12, 12–10c; 13, 13–6c; 14, 14–11c; 15, 15–15c; 16, 16–16c; 17, 17–5; 18, 18–7; 19, 19–12; 20, 20–13; 21, 21–17; 22, 22–18; 23, 23–5b; 24, 24–12b; 25, 25–17b; 26, 26–17b; 27, 27–18b; 28, 28–5c; 29, 29–12c; 30, 30–17c; 31, 31, 17c; 32, 32–18c; 33, 33–9; 34, 34–9b; 35, 39,9c.

In order to better evaluate the correlations among manna samples, the data concerning sugar content and yeast/bacterial contamination were processed by PCA analysis. The biplot illustrated in Figure 2 highlights the distribution of the different manna samples in relation to sugar composition, microbial concentrations of bacteria (GM17 and DWA-b) and yeast population (TGY and DWA-y). For the F1 factor, there is a positive correlation between sugar content and the values of microbial counts detected on TGY, DWA-y, and DWA-b, while for F2 factor, the correlation concerns only GM17. Some samples of manna (1–10, 2–6, 6–16, 7–10b, 12–10c, 13–6c, 14–11c, 15–15c, 21–17, 22–18, 26–17b, 30, 17c, 32–18c, 34–9b, and 35–9c) clustered into one main group that was statistically correlated with TGY, DWA-y, DWA-b, and sugar concentrations. On the other quadrant of biplot, manna samples (4–11, 17–5, 18–7, 29–12c, 31–17 and 33–9) were associated to GM17.

## Discussion

The aim of the present study was to evaluate the microbial communities hosted on manna samples extracted by different cultivars of *Fraxinus angustifolia* planted in the Madonie area (Palermo province). We also investigated sugar content and some physicochemical parameters characterizing different samples. The work was born from the need of the producers of manna consortia in the above mentioned area that have turned to the University of Palermo with a specific request: to characterize the manna products for cultivable microorganisms.

Manna represent very traditional food products obtained by expert hands and without addition of chemical preservatives, thus manna still retains a natural image as high-quality food and/or food adjuvant. Considering this, an increase in manna consumption could result in the increase in demand from novel consumers interested in the re-discovery of traditional food products (Souza et al., 2006; Settanni and Moschetti, 2014).

In order to better characterize the microbial stability of manna, the microbial populations and sugar content were analyzed in this study. The microorganisms present in different types of manna products (*cannolo* and *rottame*) might provide new information regarding the hygienic quality and potential application of these traditional foods. Furthermore, a deep investigation of microorganisms hosted by manna and associated with production environment might improve quality and shelf-life of final products. To this purpose, several manna samples belonging to different commercial categories were collected and subjected to the analysis of bacteria, yeasts and filamentous fungi, and physical-chemical parameters. To our knowledge, there is currently no published data on this topic, and the only few papers available on manna products focused on its chemical composition.

Concerning manna sugar content, no detailed/specific works on Arabian samples showed them to contain sucrose, melezitose, and trehalose. Our data suggested a high content of mannitol, mannotriose, and sucrose for both *cannolo* and *rottame* saples. A positive correlation between UR% and a_w_ parameters was observed.

According to the other high content sugary foods (Snowdon and Cliver, 1996; Feás et al., 2010; Sinacori et al., 2014), the microbial loads, although variable, are generally < 10^3^cfu/g. This is due to their high content of sugar, which creates stressful conditions for the growth and survival of non-osmo-tolerant microorganisms (Iurlina and Fritz, 2005). Manna products are food matrices with a sugar content of about 85% (w/w). However, several microbial isolates were collected from the samples analyzed in this study.

As reported by Sinacori et al. (2014) for honey, yeasts on high-sugary foods were mainly non-*Saccharomyces*, while the group of bacteria included several *Bacillus* species (Iurlina and Fritz, 2005; Alippi and Reynaldi, 2006; Sinacori et al., 2014). The results of our study followed the trend generally reported for other sugary foods. Presumptive LAB were not detected in any sample and only two samples turned out positive for the presence of *Enterobacteriaceae* family members, whereas the presumptive osmophilic bacteria were identified as *B. amyloliquefaciens, B. halotolerans, B. mojavensis, B. safensis, B. subtilis, B. tequilensis*, and *B. vanilla*. Most of these species are endophytic bacteria and are antagonist of fungi pathogen of plants (Bacon and Hinton, 2002), whereas *B. safensis*, that was *first* isolated in spacecraft, colonizes habitats with stringent condition for the survival of some microorganisms (Satomi et al., 2006; Lateef et al., 2015). Furthermore, within the bacteria found on DWA plates, *Clavibacter michiganensis*, and *four* species of *Staphylococcus* spp. were also identified. Staphylococci are widespread in nature and are found consistently on birds (Place et al., 2002), therefore, their presence in manna depends on the environmental contamination, as well as to the production process. *Staphylococcus epidermidis, St. haemolyticus*, and *St. warneri* are considered unsafe because some strains showed decreased susceptibility to vancomycin (Center et al., 2003), in contrast to *St. succinus* subsp. *casei* (Place et al., 2002). Although, at low levels clostridia were observed; similar findings were previously reported for honey (Sinacori et al., 2014).

In the present study, yeasts were the most abundant microbial populations and a total of six species were identified: *Candida aaseri, Candida lactis-condensi, Citeromyces matritensis, Lachancea termotolerans, Saccharomyces cerevisiae*, and *Zygosaccharomyces bailii*. The species *L. termotolerans* was the most frequently isolated. *L. thermotolerans*, like all non-*Saccharomyces* yeasts, is a widespread and applied cosmopolitan species as a starter in oenology (Hranilovic et al., 2018). It is a yeast that is able to positively influence the sensorial profile of the wine. In winemaking environments, it is to be considered a robust starter as it resists up to 13.6% (v/v) of ethanol. This species is usually applied as a starter culture to ferment several alcoholic beverages and mainly in co-fermentation with *Saccharomyces cerevisiae* to improve the overall quality of wine (Gobbi et al., 2013). Several applications of *L. termotolerans* are also reported as a starter to produce beer (Domizio et al., 2016).

Generally, *L. thermotolerans* is not known in literature as a yeast capable of resisting high sugar concentrations. Manna, like honey and other sugar matrixes, is characterized by low water activity caused by a high concentration of sugar. In literature, it is known that different species of yeasts are osmotolerant and mainly belong to the following genera: *Candida, Metschnikowia, Millerozyma* and *Zigosaccharomyces* (Kurtzman et al., 2011). The isolates of *L. thermoltolerans* found on the manna represent a particular case, but given the enormous adaptation of this species of yeast to different habitats (Hranilovic et al., 2017), this work could provide a better starting point to further study the complexity of the structure of the population, ecology and evolution of this non-*Saccharomyces* yeast.

*Citeromyces matritensis* is a yeast generally found in sugar syrups (Marvig et al., 2014). *Zygosaccharomyces bailii* is a spoilage yeast, known for its extreme resistance to preservatives and ability to grow in excess of legally-permitted concentrations of preservatives (Stratford et al., 2013), while *Candida aaseri* is a novel species, never detected in food matrices (Brandt and Lockhart, 2012).

Filamentous fungi included five genera: *Cladosporium, Mucor, Aspergillus, Alternaria*, and *Penicillium*. All genera are considered common contaminants of honey and of other foods due to their xerophilic characteristics (Nasser, 2004; Kacániová et al., 2012; Yoder et al., 2013).

## Conclusions

This work represents the first investigation on the microbial ecology of manna. Despite the stringent conditions due to high sugar content of manna, osmophilic bacteria, yeasts, and fungi filamentous were found in several samples. Plate counts on manna samples after the enrichment procedures, showed the presence of these microorganisms at high levels, confirming their survival in such stressing conditions in a viable and cultivable form.

However, the microbial ecology of manna was mainly represented by *Bacillus* and non-*Saccharomyces* yeast genera. The presence of several contaminant microorganisms, belonging to *Staphylococcus* spp., *Zygosaccharomyces*, and *Candida* genera, were also revealed. To this purpose, higher attention should be paid to the microbiological safety of manna as food.

Although more studies are needed, our results suggest that the strains of *L. thermotolerans* isolated in this study might be evaluated *in situ* for their potential to act as starters in single or in multi-combination for food applications. Further investigations should be carried out to analyze the presence and cell densities of the potential pathogenic microorganisms vectored by manna.

## Author Contributions

RS has been involved in the sampling of vegetable matrices. RGa, AA, FC, GM, RGu, and NF contributed with laboratory work, data analyses, and text writing. AT performed all the chemico-physical analyses.

### Conflict of Interest Statement

The authors declare that the research was conducted in the absence of any commercial or financial relationships that could be construed as a potential conflict of interest.

## Footnotes

^1^http://www.ncbi.nlm.nih.gov

^2^http://www.ezbiocloud.net/eztaxon
