# Supplementary material for: Insights Into the Cultivable Microbial Ecology of “Manna” Ash Products Extracted From Fraxinus angustifolia (Oleaceae) Trees in Sicily, Italy
Source: Front Microbiol. 2019 May 21;10:984. doi: 10.3389/fmicb.2019.00984 (PMC6536662; doi:10.3389/fmicb.2019.00984)
Supplement: Supplementary file 2 [file Table_2.docx]

**Table S2** Microbial loads (CFU/g or mL) of manna samples after the enrichment procedures (5d of incubation)

| **Sample code** | **Source** | **Media** | | | | | | | |
| --- | --- | --- | --- | --- | --- | --- | --- | --- | --- |
|  |  | **Bacteria** | | | | | **Yeasts** | | **FF** |
|  |  | PCA | GM17 | DWA-b | VRBGA | RCM^a^ | TGY | DWA-y | PDA |
| 1-10 Manna | Cannolo | n.d. | n.d. | n.d. | < 1^a^ | 0.00 | n.d. | 8.25 ± 0.05^a^ | n.d. |
| 2-6 Manna | Cannolo | n.d. | n.d. | n.d. | < 1^a^ | n.d. | n.d. | n.d. | < 2^a^ |
| 3-8 Manna | Cannolo | n.d. | n.d. | n.d. | < 1^a^ | 0.00 | n.d. | n.d. | < 2^a^ |
| 4-11 Manna | Cannolo | n.d. | < 1^a^ | < 2^a^ | < 1^a^ | 0.00 | n.d. | n.d. | < 2^a^ |
| 5-15 Manna | Cannolo | n.d | n.d | n.d | < 1^a^ | n.d. | n.d. | n.d. | < 2^a^ |
| 6-16 Manna | Cannolo | n.d. | n.d. | n.d. | n.d. | 0.00 | n.d. | n.d. | < 2^a^ |
| 7-10b Manna | Cannolo | n.d. | n.d. | < 2^a^ | < 1^a^ | 0.00 | < 2^a^ | n.d. | < 2^a^ |
| 8-6b Manna | Cannolo | n.d. | n.d. | n.d. | < 1^a^ | 0.00 | n.d. | n.d. | < 2^a^ |
| 9-11b Manna | Cannolo | n.d. | n.d. | n.d. | < 1^a^ | n.d. | n.d. | n.d. | < 2^a^ |
| 10-15b Manna | Cannolo | n.d. | n.d. | < 2^a^ | n.d. | 0.00 | n.d. | n.d. | < 2^a^ |
| 11-16b Manna | Cannolo | n.d. | n.d. | n.d. | < 1^a^ | 0.00 | n.d. | n.d. | < 2^a^ |
| 12-10c Manna | Cannolo | n.d. | n.d. | n.d. | < 1^a^ | n.d. | n.d. | n.d. | < 2^a^ |
| 13-6-1c Manna | Cannolo | n.d. | n.d. | n.d. | < 1^a^ | 0.00 | n.d. | n.d. | < 2^a^ |
| 14-11c Manna | Cannolo | < 2^b^ | n.d. | n.d. | < 1^a^ | 0.00 | < 2^a^ | < 2^b^ | < 2^a^ |
| 15-15c Manna | Cannolo | < 2^b^ | n.d. | < 2^a^ | < 1^a^ | 0.00 | n.d. | n.d. | < 2^a^ |
| 16-16c Manna | Cannolo | n.d. | n.d. | n.d. | < 1^a^ | 0.00 | n.d. | n.d. | n.d. |
| 17-5 Manna | Rottame | 6.10 ± 0.40^a^ | n.d. | n.d. | < 1^a^ | 0.00 | n.d. | < 2^b^ | n.d. |
| 18-7 Manna | Rottame | n.d. | n.d. | < 2^a^ | < 1^a^ | 0.00 | n.d. | n.d. | < 2^a^ |
| 19-12 Manna | Rottame | n.d. | n.d. | n.d. | < 1^a^ | n.d. | n.d. | n.d. | < 2^a^ |
| 20-13 Manna | Rottame | n.d. | n.d. | n.d. | n.d. | n.d. | n.d. | n.d. | < 2^a^ |
| 21-17 Manna | Rottame | n.d. | n.d. | n.d. | < 1^a^ | n.d. | n.d. | n.d. | n.d. |
| 22-18 Manna | Rottame | n.d. | n.d. | n.d. | < 1^a^ | 0.00 | n.d. | n.d. | < 2^a^ |
| 23-5b Manna | Rottame | < 2^b^ | n.d. | n.d. | < 1^a^ | n.d. | n.d. | n.d. | < 2^a^ |
| 24-7b Manna | Rottame | < 2^b^ | n.d. | n.d. | < 1^a^ | 0.00 | n.d. | n.d. | n.d. |
| 25-17b Manna | Rottame | n.d. | n.d. | n.d. | < 1^a^ | n.d. | n.d. | n.d. | n.d. |
| 26-17b Manna | Rottame | < 2^b^ | n.d. | < 2^a^ | n.d. | n.d. | n.d. | < 2^b^ | < 2^a^ |
| 27-18b Manna | Rottame | n.d. | n.d. | n.d. | < 1^a^ | n.d. | n.d. | n.d. | < 2^a^ |
| 28-5-1c Manna | Rottame | n.d. | n.d. | n.d. | < 1^a^ | n.d. | n.d. | n.d. | < 2^a^ |
| 29-12d Manna | Rottame | < 2^b^ | n.d. | < 2^a^ | < 1^a^ | 0.00 | < 2^a^ | n.d. | < 2^a^ |
| 30-13c Manna | Rottame | < 2^b^ | n.d. | n.d. | < 1^a^ | 0.00 | n.d. | n.d. | n.d. |
| 31-17d Manna | Rottame | < 2^b^ | n.d. | n.d. | < 1^a^ | n.d. | n.d. | n.d. | n.d. |
| 32-18c Manna | Rottame | < 2^b^ | n.d. | n.d. | < 1^a^ | n.d. | n.d. | n.d. | < 2^a^ |
| 33-9 Manna | Liquid | n.d. | < 1^a^ | n.d. | < 1^a^ | 0.00 | n.d. | n.d. | n.d. |
| 34-9g Manna | Liquid | n.d. | n.d. | n.d. | < 1^a^ | 0.00 | n.d. | n.d. | n.d. |
| 35-9-1b Manna | Liquid | n.d. | n.d. | n.d. | < 1^a^ | 0.00 | n.d. | n.d. | n.d. |
| Statistical significance^b^ | | *** | NS | NS | NS | ND | NS | *** | NS |

^a^ As estimated by MPN.

*Abbreviations*: FF, filamentous fungi; PCA, plate count agar for total mesophilic bacteria; GM17, glucose M17 added with cycloeximide for lactic acid bacteria counts; DWA-b, De Whalley agar added with cycloeximide for osmophilic bacteria counts; VRBGA, violet red bile glucose agar for *Enterobacteriaceae* counts; RCM, reinforced clostridial medium; TGY, tryptone glucose yeast extract agar added with chloramphenicol for total (osmophilic and osmotolerant) yeasts; DWA-y, De Whalley agar added with chloramphenicol for osmotolerant yeast counts; PDA, potato dextrose agar for filamentous fungi counts; n.d., not determined since the samples showed viable counts of microorganisms without the enrichment procedures as reported in Table 2.

Results indicate mean values with standard deviation

Data within a column followed by the same letter are not significantly different according to Tukey's test.

^b^P value:***, P < 0.001 ; NS, not significant; ND, not determined.
